# Motor Axon Synapses on Renshaw Cells Contain Higher Levels of Aspartate than Glutamate

**DOI:** 10.1371/journal.pone.0097240

**Published:** 2014-05-09

**Authors:** Dannette S. Richards, Ronald W. Griffith, Shannon H. Romer, Francisco J. Alvarez

**Affiliations:** 1 Department of Neuroscience, Cell Biology and Physiology, Wright State University, Dayton, Ohio, United States of America; 2 Department of Physiology, Emory University, Atlanta, Georgia, United States of America; Virginia Tech Carilion Research Institute, United States of America

## Abstract

Motoneuron synapses on spinal cord interneurons known as Renshaw cells activate nicotinic, AMPA and NMDA receptors consistent with co-release of acetylcholine and excitatory amino acids (EAA). However, whether these synapses express vesicular glutamate transporters (VGLUTs) capable of accumulating glutamate into synaptic vesicles is controversial. An alternative possibility is that these synapses release other EAAs, like aspartate, not dependent on VGLUTs. To clarify the exact EAA concentrated at motor axon synapses we performed a quantitative postembedding colloidal gold immunoelectron analysis for aspartate and glutamate on motor axon synapses (identified by immunoreactivity to the vesicular acetylcholine transporter; VAChT) contacting calbindin-immunoreactive (-IR) Renshaw cell dendrites. The results show that 71% to 80% of motor axon synaptic boutons on Renshaw cells contained aspartate immunolabeling two standard deviations above average neuropil labeling. Moreover, VAChT-IR synapses on Renshaw cells contained, on average, aspartate immunolabeling at 2.5 to 2.8 times above the average neuropil level. In contrast, glutamate enrichment was lower; 21% to 44% of VAChT-IR synapses showed glutamate-IR two standard deviations above average neuropil labeling and average glutamate immunogold density was 1.7 to 2.0 times the neuropil level. The results were not influenced by antibody affinities because glutamate antibodies detected glutamate-enriched brain homogenates more efficiently than aspartate antibodies detecting aspartate-enriched brain homogenates. Furthermore, synaptic boutons with ultrastructural features of Type I excitatory synapses were always labeled by glutamate antibodies at higher density than motor axon synapses. We conclude that motor axon synapses co-express aspartate and glutamate, but aspartate is concentrated at higher levels than glutamate.

## Introduction

The release of acetylcholine from motor axons at the mammalian neuromuscular junction (NMJ) has been established for more than 75 years [1], but recent studies suggest that additional neurotransmitters, in particular excitatory amino acids (EAAs) like glutamate, might be co-released from motoneuron synapses both in the periphery and centrally. High levels of glutamate, EAA transporters and AMPA/NMDA receptors have been detected in motor end-plates [2]–[4] and significant actions of glutamate receptors on NMJ cholinergic neurotransmission have been described. For example, activation of presynaptic metabotropic glutamate receptors modulates acetylcholine neurotransmitter release at the NMJ [5], [6] and postsynaptic NMDA receptor-mediated nitric oxide release regulates acetylcholinesterase activity [7]. However, motor axon postsynaptic actions on normal mammalian muscles are fully blocked by nicotinic acetylcholine receptor antagonists and a contribution from NMDA/AMPA receptors to postsynaptic end-plate currents is not commonly observed. Nevertheless, NMDA/AMPA receptor responses can be induced experimentally after muscle dennervation and re-innervation with glutamatergic axons [8], [9].

Motoneuron axons also extend collaterals inside the spinal cord and establish synapses with Renshaw cells, an interneuron that provides feedback inhibition to the same motoneurons [10], [11]. Similar to the NMJ, motor axon actions on Renshaw cells were also found to be cholinergic at first [10], [12], a finding that, at the time, confirmed Dale's principle for the equivalence of neurotransmitter release in all synaptic boutons from single axons (Eccles, 1976). Acetylcholine receptor antagonists, however, did not fully inhibit the postsynaptic actions of motor axons on Renshaw cells. In the original studies it was argued that this was due to relatively low concentrations of antagonists inside synaptic clefts during *in vivo* pharmacological experiments [10], [12]. Later, *in vitro* studies (spinal cord slices or whole neonatal spinal cords) also failed to fully inhibit Renshaw cell-mediated disynaptic recurrent inhibition of motoneurons or motor axon excitatory postsynaptic currents (EPSCs) on Renshaw cells with acetylcholine [13], [14]. In this case receptor antagonists were bath applied to either isolated spinal cords or spinal cord slices, an experimental situation believed to result in better saturation of postsynaptic receptors by antagonists. More recent analyses in neonatal mouse *in vitro* spinal cord preparations demonstrated that motor axon evoked EPSPs and EPSCs on Renshaw cells display various components mediated respectively by nicotinic, NMDA and AMPA receptors [15]–[18] and that, similar to the NMJ, glutamate-immunoreactivity is enriched in motor axon synapses on Renshaw cells [17]. The presence of significant NMDA receptor postsynaptic currents could explain the relatively longer time course of motor axon synaptic actions on Renshaw cells compared to muscle, a fact that puzzled investigators since it was first described [10], [19]. Furthermore, late Renshaw cell discharges in response to motor axon input were shown to be NMDA-dependent in the neonatal spinal cord [15].

Despite these advances, the exact mechanisms used by motor axons to co-release acetylcholine and possibly glutamate remained unclear. Most studies agree that vesicular glutamate transporters (VGLUTs) are not co-localized with vesicular acetylcholine transporters (VAChT) at motor axon synapses contacting Renshaw cells [17], [18], [20], [21]. Two studies using intracellular fills of single motor axons in neonates raised the possibility that, contrary to Dale's principle, motor axons could traffic VGLUTs (specifically VGLUT2) and VAChT to different axon collaterals and thus release different neurotransmitters at different synapses [18], [20], however, this could not be confirmed in other studies using similar methods in adult cats [21] or using bulk labeling of motor axons from ventral roots in neonatal rodent spinal cords [17]. Whether motoneurons express VGLUT2 is also controversial. Some *in situ* hybridization studies detected VGLUT2 mRNA in motoneurons [18], [20], [22], but others did not [23]–[25]. Given that VGLUT expression is necessary for the synaptic release of glutamate [26]–[28], it is of concern that VGLUTs cannot be consistently demonstrated in motoneurons or their synapses. One possibility is that motoneurons express very low levels of VGLUT2, perhaps close to the threshold for detection. This would explain the disparity of results using more or less stringent labeling conditions or criteria. But, given the relationship between VGLUT2 expression levels, vesicular glutamate concentration and postsynaptic action [29], it is difficult to reconcile low levels of VGLUT2 with consistent motor axon activation of NMDA receptors on Renshaw cells.

One alternative possibility is that other compounds capable of activating NMDA and/or AMPA receptors are released from the motor axons through VGLUT-independent mechanisms. One possibility is that these synapses are in part aspartaergic. Aspartate has similar affinity for NMDA receptors as glutamate and generates similar NMDA currents [30], but its transport into synaptic vesicles is independent of VGLUTs [26], [31]–[34]. To clarify the exact EAA content of motor axon synapses on Renshaw cells we analyzed their relative enrichment in glutamate and aspartate. Because the large majority (>80%) of VAChT-immunoreactive (-IR) synapses on Renshaw cells originate from motor axons and few (<5%) from other sources [35], we identified motor axon synapses with dual preembedding immunolabeling to detect VAChT-IR synapses (using immunogold silver) on calbindin-IR Renshaw cell dendrites (identified with immunoperoxidase). These dual imunolabeled sections were then tested for glutamate or aspartate content using postembedding colloidal immunogold methods. Quantification of these electron microscopy triple immuolabeled preparations suggested that aspartate is more highly and consistently enriched than glutamate in motor axon terminals on Renshaw cells.

## Materials and Methods

### Animals

All animal procedures were done in accordance with Wright State University's Laboratory Animal Use Committee and NIH guidelines and were approved by Wright State University Institutional Animal Care and Use Committee (IACUC) committee. For electron microscopy, C57/bl mice were anesthetized with pentobarbital (Euthasol 50–70 mg/kg i.p.) and perfused transcardially, first with a cold vascular rinse (0.01 M phosphate buffer with 137 mM NaCl, 3.4 mM KCl, and 6 mM NaHCO3, pH 7.3) followed by 2.5% glutaraldehyde (Electron Microscopy Sciences, Hatfield, PA) and 1.0% paraformaldehyde in 0.1 M phosphate buffer (PB). The spinal cords were then removed, post-fixed in the same fixative for 4 hours and cut in 100 µm thick vibratome sections.

### Pre-embedding electron microscopy immunolabeling for calbindin and VAChT

The sections were pretreated with 1% NaBH_4_ (Sigma-Aldrich, St. Louis, MO) in PBS and blocked for 30 minutes in 10% normal donkey serum (Jackson ImmunoResearch, West Grove, PA) diluted in PBS and then incubated in a goat anti-VAChT polyclonal antibody (Millipore, Temecula, CA; cat. number: AB1578, lot: 602021588) diluted 1∶1,000 in PBS overnight at room temperature. Sections were then incubated in a biotinylated-SP anti-goat secondary antibody (Jackson Immunoresearch) diluted 1∶100 and then in streptavidin coupled to 1.4 nm gold particles (Nanoprobes, Yaphank, NY) at a 1∶50 dilution (both in PBS). The tissue was treated with 50 mM glycine in PBS and bovine serum albumin at 1.0% (Sigma-Aldrich) in PBS and then rinsed briefly in nano-distilled H_2_O (*nd*H_2_O) before silver intensification with an HQ Silver Enhancement Kit (Nanoprobes). Developing reactions to generate silver particles on VAChT-immunoreactive (-IR) sites lasted from 2 to10 minutes. The silver reaction product was stabilized with 0.05% gold chloride solution followed by rinses in 0.2 M tris maleate buffer and a brief incubation in 3% sodium thiosulfate solution to dissolve any remaining unbound silver. Following these rinses the tissue sections were again incubated overnight in a new primary antibody solution containing rabbit anti-calbindin (Swant, Bellinzona, Switzerland; cat. number: CB-38a, lot: 9.03), diluted 1∶500 in PBS to label Renshaw cells and their processes. To reveal calbindin-immunoreactivity we used anti-rabbit ABC-peroxidase standard Vector kits (Vector Labs Inc., Burlingame, CA). Peroxidase-labeled immunoreactive sites were revealed with 0.02% diaminobenzidine (DAB) and 0.01% hydrogen peroxide in PBS. The reactions were carried out for 5–10 minutes at room temperature.

### Cryosubstitution and Lowicryl embedding

Immunolabeled sections were cryoprotected in an ascending series of glycerol solutions diluted in 0.1 M PB (30 minute incubations at 10%, 20%, and 30% strength), followed by overnight incubation in 30% glycerol. The following day the sections were freeze-slammed using the “copper-mirror” technique in a Leica MM80 apparatus. The frozen sections were then placed at -80°C in a cryosubstitution chamber and rinsed 4 times in methanol for 30 minutes each. This was followed by an ascending series of methanol∶Lowicryl solutions at −50°C and two changes into fresh 100% Lowicryl where it remained overnight. The tissue was flat-embedded between teflon-coated glass coverslips and placed under ultraviolet light for resin polymerization at the following temperature schedule: −50°C for 49 hours, raised to 0°C over a time of 5 hours, held at 0°C for 72 hours, raised to 20°C over a time of 10 hours, followed by a 24 hours at 20°C.

### Colloidal gold post-embedding with aspartate and glutamate antibodies

Areas of interest containing calbindin-IR Renshaw cells were identified under the light microscope in flat-embedded section wafers and then cut out and placed on EM beam resin capsules for ultramicrotomy. Ultrathin sections (∼70–90 nm thick, gold-silver interference contrast) were obtained and collected on 200 mesh nickel grids coated with Formvar (Electron Microscopy Sciences). The grids were then processed for postembedding colloidal gold immunostaining. First, they were pre-incubated in 10% normal goat serum (Vector) and then incubated overnight at 4°C in a primary antibody solution containing either rabbit anti-glutamate (0.17 µg/ml & 0.034 µg/ml, Alpha Diagnostics International, San Antonio, TX; Cat.#: GLM21-S, lot:40128S) or rabbit anti-aspartate (0.36 µg/ml and 0.18 µg/ml, Biogenesis Ltd., Poole, UK; Cat. number 0770-0004, lot:A980421) diluted in Tris Buffer Saline with 0.05% Tween (TBS-Tw) at a pH of 7.6. The next day the grids were rinsed in TBS-Tw pH 7.6 followed by a rinse in TBS-Tw pH 8.2 and incubated in a secondary antibody solution containing anti-rabbit antibodies conjugated to 10 nm colloidal gold particles (British BioCell International (BBI), Cardiff, UK) at 1∶25 dilution in TBS-Tw pH 8.2 for 2 hours. Following this incubation, grids were rinsed in nanopure distilled water (*nd*H_2_O) and the ultrathin sections were then contrasted using 1% uranyl acetate solution and standard Reynold's lead citrate solution.

### Electron microscopy image capturing and analysis

Contrasted and immunolabeled ultrathin sections were examined in a Phillips 208S electron microscope at 70 kV. Images were obtained photographically (EM 4×5 negatives) and the negatives scanned at 1200 dpi (Flextight 848; Imacom, Copenhagen, Denmark). Micrographs were obtained from 4 to 6 ultrathin sections from different regions in the Renshaw area in each of three different mice analyzed. Scanned images containing VAChT-IR boutons were identified by their immunogold immunoreactivity and classified as belonging to motor axons if they established a synapse on a calbindin-IR Renshaw cell soma or dendrite or classified as a C-bouton (originated in cholinergic interneurons) if the synapse was on a motoneuron cell body and displayed a submembrane cistern [36]. Other VAChT-IR terminals in the neuropil have more uncertain origins and were not included in the analyses.

Quantitative analyses were carried out using Image Pro Plus (ver. 5.1; Media Cybernetics, Silver Spring, MD). The area of axoplasm from which neurotransmitters could be loaded into synaptic vesicles was determined by measuring the area of the bouton and subtracting the area occupied by mitochondria. Then the number of colloidal gold particles inside the bouton, but outside mitochondria, was counted and a density calculated (number of gold particles per 1 µm^2^). Average densities of gold particles were estimated for each animal or the whole sample of VAChT-IR boutons contacting calbindin-IR Renshaw cells dendrites and VAChT-IR boutons contacting motoneurons (mostly C-boutons). To determine if there was immunogold enrichment inside VAChT-IR boutons above average neuropil staining, 1 µm^2^ regions of interest (ROIs) were similarly analyzed in the neuropil. Neuropil ROIs were collected from the regions at the four corners of the scanned negatives. Similar to the boutons, areas associated with mitochondria and their associated gold particles were subtracted from the density calculation. Any neuropil test squares covering areas of the photograph that contained VAChT-IR synapses, artifacts or grid bars were excluded.

### Statistical comparisons

When possible paired groups of data were compared using standard t-tests and multiple groups with standard one-way ANOVA followed by pair-wise comparisons among the groups using a Tukey test. However, data were frequently not normally distributed. This was usually the case in samples with low levels of immunolabeling and skewed data points or in samples with several estimates at zero. In these cases we used non-parametric tests: Mann-Whitney Rank-Sum test for comparing two groups and Kruskal-Wallis ANOVA on Ranks for more than two groups followed by post-hoc Dunn's test for pair-wise comparisons. Statistical significance was set at p<0.05. All statistics were run in Sigma-Stat 3.1 (Jandel Corporation, Chicago, IL).

### Antibody specificity

#### Aspartate and glutamate antibodies

**Figure 1:**
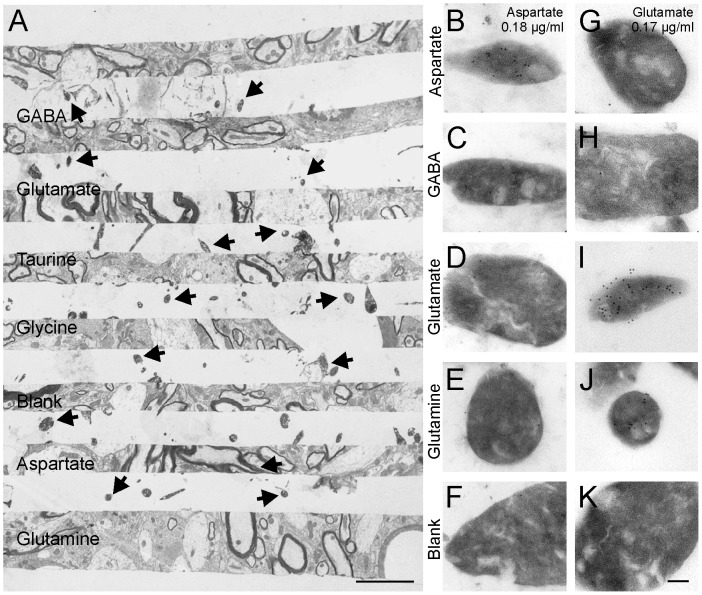
Aspartate and glutamate antibody specificity. **A**, Layers of amino acid enriched brain homogenates embedded between brain tissue sections. Brain homogenate islands (arrows) contain different glutaraldehyde-fixed amino acids. Tissue sections used as spacers are all 2 µm thick except for the last one which is 4 µm and used for sequence orientation. **B–F**, Gold immunolabeling of islands enriched with aspartate, GABA, glutamate and glutamine using the aspartate antibody at 0.18 µg/ml concentration. As controls some brain homogenates were not embedded with any amino acids (blank). Only islands embedded with aspartate (B) contained dense immunogold labeling. **G–K**, Immunolabeling with glutamate antibody at 0.17 µg/ml. Islands embedded with glutamate (I) contained dense immunogold labeling. Weaker immunolabeling was observed in glutamine islands (J). Islands embedded with aspartate (G), GABA (H) glycine (not shown) or taurine (not shown) did not contain any significant immunogold labeling, nor did the blank or control islands lacking any amino acids (K). Scale bars, 3 µm in A; 1 µm in J.

The rabbit aspartate polyclonal antibody (Biogenesis) was raised against a glutaraldehyde conjugate of aspartate and bovine thyroglobulin while the rabbit glutamate polyclonal antibody (Alpha Diagnostics International) was raised against a glutaraldehyde conjugate of glutamate and hemocyanin. The specificity of the antibodies was tested against various glutaraldehyde fixed amino acids using resin sandwiches kindly provided by Dr. O. P. Ottersen (University of Oslo, Norway). The production of test sandwiches is described elsewhere [37]. Layers with brain sections were separated by layers of brain homogenate “islands” contained the following glutaraldehyde-fixed amino acids: aspartate, GABA, glutamate, glutamine, glycine, taurine, and no amino acids (Fig. 1). Electron microscopy grids containing these test sections were immunolabeled with colloidal gold particles as described above. High magnification images (100,000x) were taken of the amino acid enriched brain homogenate “islands” with a digital camera coupled to the electron microscope (Gatan, Bio Scan II, Pleasonton, CA) and the density of gold particles calculated.

#### Calbindin antibodies

The rabbit polyclonal antibody used in electron microscopy experiments was raised against rat recombinant calbindin D28k and detects a single 28 kDa band in western blots of brain homogenates of tissue originating from several species including rat, rabbit, guinea pig, mouse, chicken and zebrafish (information provided by manufacturer: Swant, Bellinzona, Switzerland), it does not stain any structures in the spinal cord of calbindin-D28k knockout mice (Valerie C. Siembab and Francisco J. Alvarez, unpublished) and immunostains the same Renshaw cells detected with *in situ* hybridization for calbindin mRNA transcript [38]. In dual immunofluorescence preparations between this polyclonal calbindin antibody and a monoclonal antibody produced by hybridization of myeloma cells with spleen cells from mice immunized with calbindin D28K purified from chicken gut [39], both antibodies labeled the same cells (not shown).

#### VAChT antibody

We used a polyclonal antibody raised in goat against amino acids 511–530 in the C-terminus of rat VAChT (Millipore, cat. no. AB1588; lot#848850). This antibody revealed the same VAChT expressing spinal cord cells detected with *in situ* hybridization [38]. At the ultrastructural level VAChT-IR boutons on motoneurons display the morphological features of cholinergic C-boutons [36], (also see results) and VAChT-immunoreactivity using these antibodies also detected specifically synaptic varicosities on Renshaw cells originated from motor axons retrogradely labeled from the ventral root [17].

## Results

### Specificity of polyclonal aspartate and glutamate antibodies

**Figure 2:**
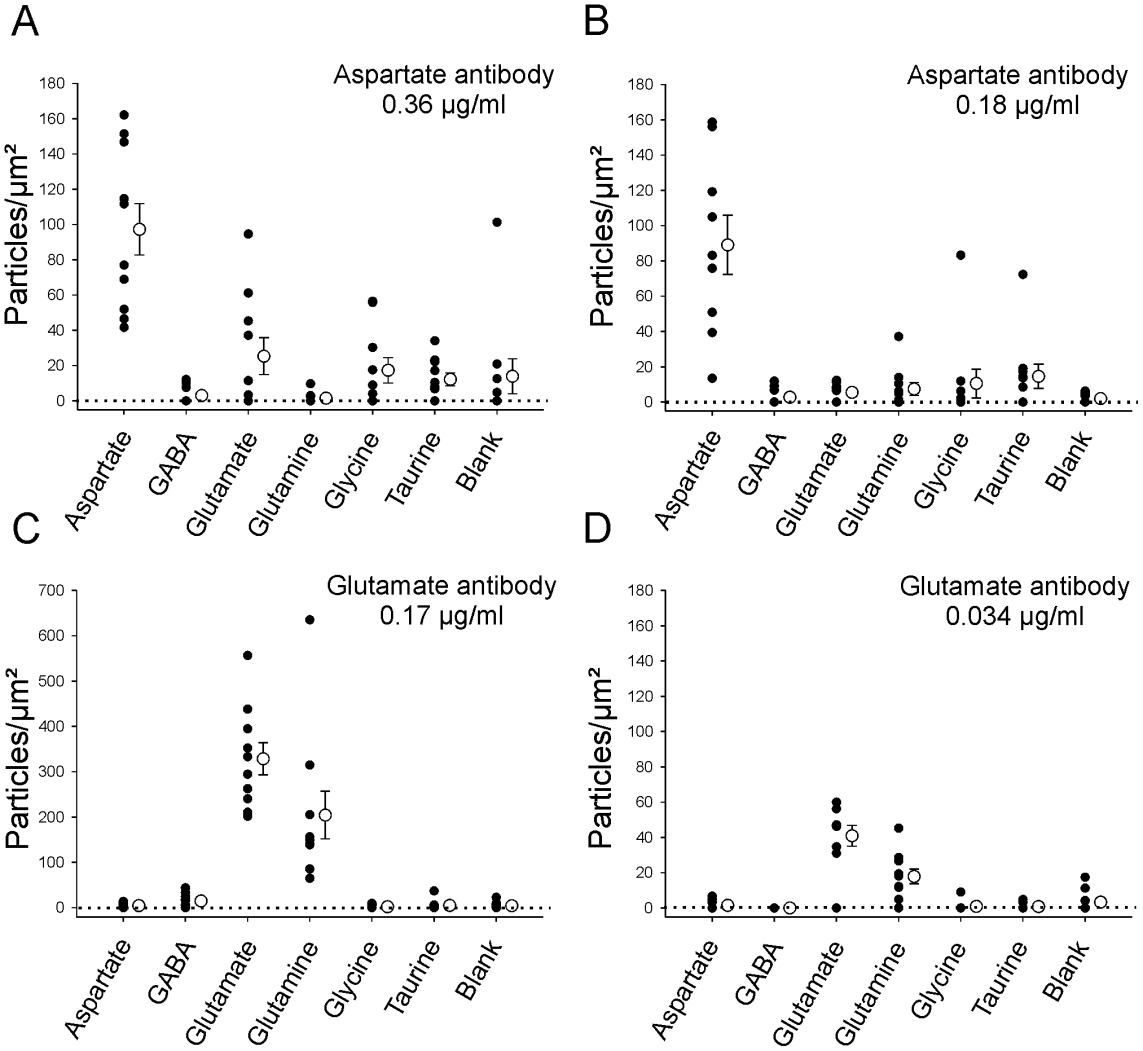
Quantitative analysis of aspartate and glutamate antibody labeling in resin sandwiches. **A, B**, Density of labeling of amino acid enriched islands at high (A) and low (B) aspartate antibody dilutions. Filled black dots indicate individual density estimates for each sampled island. White circles show averaged data. Error bars indicate ± SEM. Dotted line indicates 0 labeling. Only aspartate-enriched islands were significantly immunolabeled above the density of blank islands (background). **C, D**, Similar density plots for glutamate immunolabeling at low and high antibody dilutions. Note differences in labeling density between both antibody dilutions (different y-axis values) and also the significant labeling of glutamine islands with glutamate antibodies.

Specificity was determined on layers of brain homogenates containing various glutaraldehyde-fixed amino acids (aspartate, GABA, glutamate, glycine and taurine) or no amino acid. Brain homogenate particles (“islands”) enriched with the different amino acids were sandwiched in between layers of brain tissue (Fig. 1A) and immunolabeleld with aspartate or glutamate antibodies in post-embedding (Figs. 1B–K). Two different antibody concentrations were tested. The density of 10 nm colloidal gold particles was determined in ten islands for each amino acid conjugate, immunolabeling and antibody dilution (Fig. 2).

At both antibody dilutions, aspartate immunolabeling was higher in aspartate islands compared to other amino acids (Figs. 1B–F, 2A, B) and the differences in average density were always significant (p<0.001, Kruskal-Wallis ANOVA on Ranks, p<0.05 Post-hoc Dunn's test). The density of aspartate immunolabeling in aspartate enriched islands varied from dense to weak and covered a rather similar range at both antibody dilutions, except for a few weakly labeled islands using the lower antibody concentration (Figs. 2A,B). Average labeling density in aspartate islands was not significantly different between antibody dilutions (t-test, p = 0.756). However, a proportion of islands enriched with other amino acids showed variable aspartate immunoreactivity when the antibody was applied at high concentration. Specifically, some glutamate enriched islands displayed aspartate immunoreactivity, however, on average, aspartate immunoreactivity in glutamate islands was not statistically different from control blank brain homogenates (p = 0.426, Mann-Whitney Rank Sum test).

Glutamate immunolabeling on glutamate enriched islands was significantly higher when using the more concentrated antibody dilution (0.17 µg/ml) compared to the least concentrated (0.034 µg/ml) (t-test, p<0.001) (Figs. 1I, 2C,D). Also, glutamate immunolabeling at both dilutions was always significantly higher on glutamate enriched islands compared to any other amino acid or blank controls (p<0.001, Kruskal-Wallis ANOVA on Ranks, p<0.05 Pot-hoc Dunn's test). With the exception of glutamine, all other amino acid enriched islands were no different from blank controls. Glutamate-immunolabeling of glutamine islands was significantly higher from control at both dilutions (p<0.01, Mann-Whitney Rank Sum test). Glutamate antibodies did not show any significant cross-reactivity with aspartate at any dilution.

In conclusion, the aspartate antibodies used here are quite specific for glutaraldehyde-fixed aspartate, but at 0.36 µg/ml concentration they might sometimes weakly cross-react with glutamate. This did not occur at the 0.18 µg/ml concentration of aspartate antibody. The glutamate antibodies in contrast, do not recognize aspartate at the dilutions used, but they have significant cross-reactivity with glutamine. Signal resolution was better with 0.17 µg/ml of glutamate antibody. Finally, at similar concentrations of their respective specific antibodies the resulting immunoreactivities (particle density) were much higher for glutamate than for aspartate.

### Characteristics of VAChT and calbindin immunoreactivities in the ventral horn

**Figure 3:**
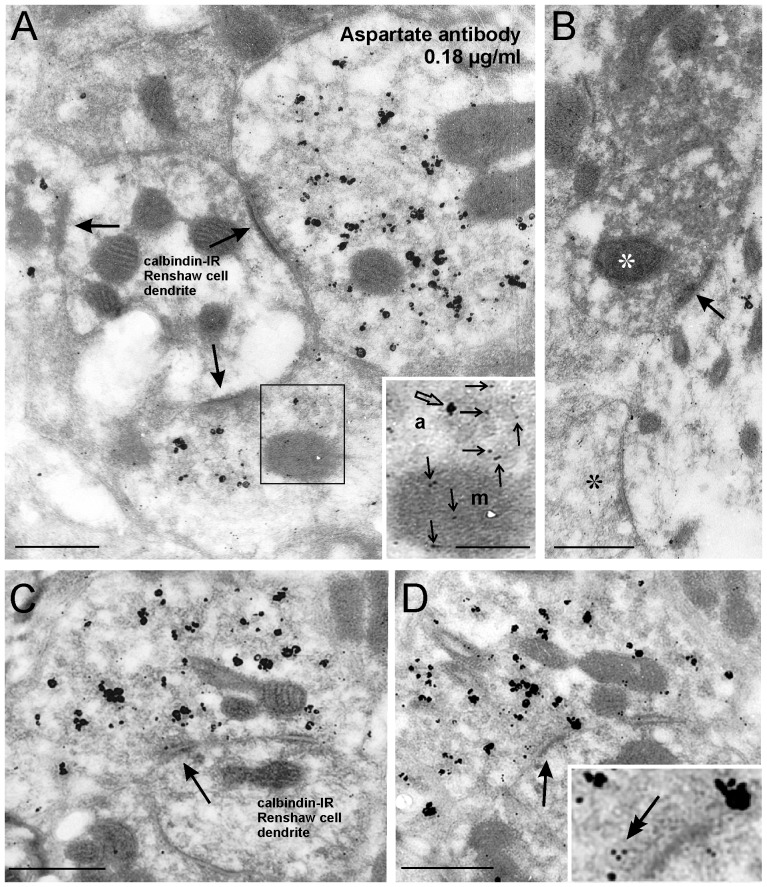
Aspartate-IR is enriched in VAChT-IR boutons presynaptic to calbindin-IR Renshaw cell dendrites. These examples were labeled with 0.18 µg/ml of aspartate antibody. **A**, Three VAChT-IR motoneuron terminals labeled with large and irregular immunogold-silver deposits (irregular black particles) make synapses (arrows) with a calbindin-IR Renshaw cell dendrite labeled with DAB. Aspartate-immunogold particles (small and uniform 10 nm size particles) are at higher density inside the three VAChT-IR synaptic boutons than in the neighboring neuropil or in the postsynaptic Renshaw cell dendrite. Inset shows at higher magnification 10 nm colloidal gold particles (arrows) in the axoplasm (a) or a mitochondria (m) from the area outlined in A. For comparison a silver deposit (VAChT-IR) is indicated with a big arrow. **B**, Aspartate-immunogold did not label inhibitory calbindin-IR Renshaw cell synaptic terminals (white asterisk). This Renshaw cell terminal establishes a synapse (arrow) with a large dendrite that contained a low density of aspartate-IR colloidal gold particles. A nearby synaptic bouton (black asterisk) contained a larger amount of colloidal gold particles. **C, D**, VAChT-IR motoneuron synaptic boutons (a synapse with a calbindin-IR Renshaw cell dendrite is indicated with an arrow) show similar levels of aspartate immunogold particles in serial sections (two sections in the series shown). Inset in D shows gold labeling inside a vesicle at the presynaptic active zone (double arrow) Scale bars are 0.5 µm in A, B, C and D; 0.25 µm in the inset in A.

**Figure 4:**
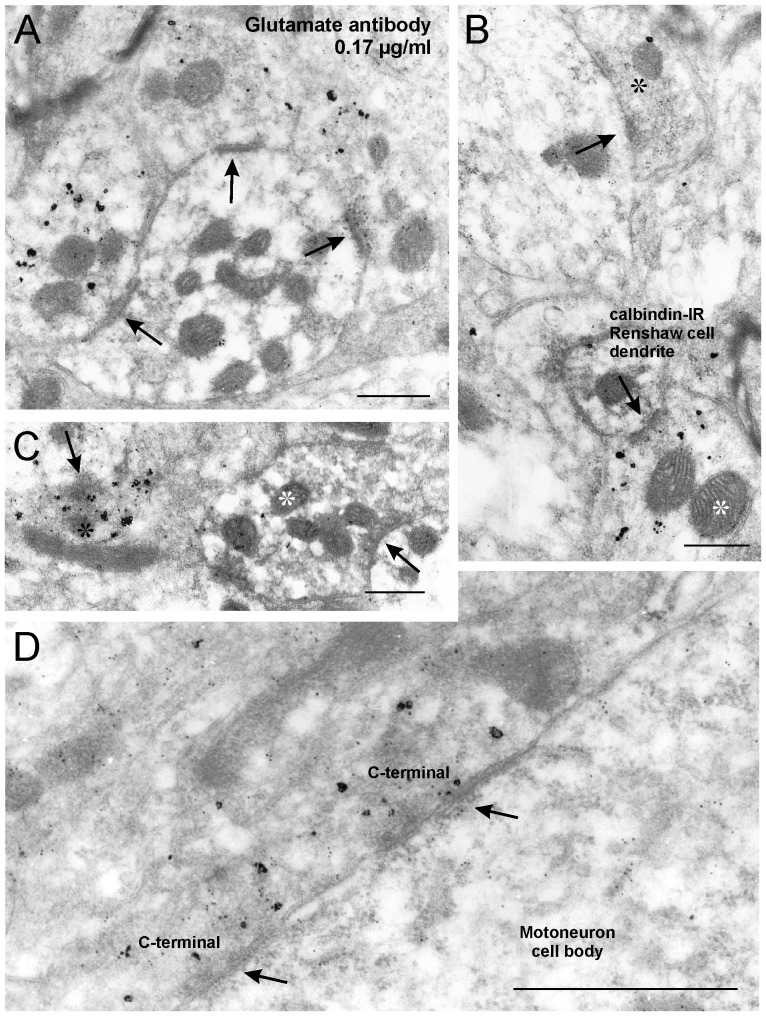
Glutamate-IR is enriched in VAChT-IR motoneuron synapses contacting Renshaw cell dendrites. Examples obtained with antibody concentration of 0.17 µg/ml. **A**, Three VAChT-IR boutons surround and make synapses (arrows) on a calbindin-IR Renshaw cell dendrite. Glutamate-IR 10 nm colloidal gold particles are enriched inside VAChT-IR motoneuron terminals compared to surrounding neuropil or calbindin-IR Renshaw dendrites. **B**, Glutamate-IR colloidal gold particles inside a VAChT-IR motoneuron synaptic bouton (white asterisk) making a synapse (arrow) with a small caliber calbindin-IR Renshaw cell dendrite and an adjacent excitatory synaptic bouton (black asterisk) establishing another synapse (arrow) on a different larger dendrite. Usually VAChT-IR synaptic boutons contain lower densities of glutamate-IR gold particles than adjacent unlabeled terminals. **C**, Calbindin-IR Renshaw cell synaptic terminal (containing DAB labeling, white asterisk) making a synapse (arrow) with an unlabeled dendrite. These inhibitory terminals contained glutamate-IR at densities similar or below neuropil labeling. A nearby VAChT-IR terminal is making a synaptic contact (arrow) with a dendrite of unknown origin and contains higher levels of glutamate IR gold particles. **D**, VAChT-IR C-boutons (silver deposits) contacting a motoneuron soma. Significant levels of glutamate -IR colloidal gold particles are found inside the C-boutons and to a lesser extent the motoneuron cell body. These synapses have a characteristic subsynaptic cistern in the postsynaptic site (arrows). Scale bars are 0.5 µm in A, B and C; 1 µm in D.

Tissue sections were dual immunolabeled in preembedding for VAChT and calbindin. Each immunoreactivity was respectively revealed with immunogold-silver (VAChT) or immunoperoxidase (calbindin). Renshaw cell area regions containing both immunomarkers were identified with light microscopy in flat embedded preparations, re-cut with ultramicrotomy and the ultrathin sections processed for postembedding aspartate or glutamate colloidal gold (10 nm) immunolabeling. Silver intensified nanogold VAChT immunoreactivity is recognized as relatively large and irregularly shaped dark silver deposits (Figs. 3 and 4). These deposits were found in cholinergic synapses contacting Renshaw cells and at a lower density in motoneuron somata and dendrites, as well as, at very high density in C-boutons (Fig. 4D), a particular type of cholinergic synapse on motoneuron cell bodies [40]. The relative density of VAChT-IR silver deposits within boutons was maximal over vesicle pools, but the absolute density varied depending on the depth at which the ultrathin section was obtained within the thickness of the 50 µm immunolabeled section. Labeling was denser at the surfaces of the section and decreased significantly after the first 5 µm from the surface of the tissue section. This is due to the relatively poor penetration of antibodies in the thick sections during pre-embedding immunostaining using immunogold-silver techniques. Terminals with very dense VAChT labeling were excluded from quantification, as it was difficult to detect 10 nm colloidal gold particles within areas containing high density silver deposits. In addition, heavily labeled terminals were typically located near the surface of the section, an area with usually worse tissue preservation and high background. VAChT-IR terminals sampled deeper in the tissue section and with weaker labeling were ideal for quantifying colloidal gold immunolabeling.

C-boutons contacting motoneuron somata were usually characterized by the presence of subsurface cisterns [36] (Fig. 4D). C-boutons originate from interneurons [41], [42] and are therefore different from cholinergic synapses on Renshaw cells that originate from motor axons [35]. Unfortunately, ultrastructural definition in these non-osmicated and triple immunolabeled sections is compromised and the presence of subsurface cisterns underneath some VAChT-IR boutons on motoneuron somata was sometimes unclear. Therefore, we cannot exclude the possibility that a few VAChT-IR synapses on motoneurons may not be C-boutons and could originate from motor axons [43].

Calbindin-IR Renshaw cell dendritic profiles were labeled with diaminobenzidine (DAB). Calbindin-IR was observed in Renshaw cell somata and dendrites (Figs. 3A,C,D and 4A,B), as well as in calbindin-IR Renshaw cell synaptic terminals (Figs. 3B, 4C). DAB labeling appears as electron dense diffuse precipitate that is hydrophobic and therefore becomes associated with lipid membranes, such as the plasma membrane or membranes of cell organelles. DAB labeling also diminishes with increasing depth in the section. Moreover, DAB electron density was generally weak in Lowicryl embedded tissue, as with cryosubstitution techniques, osmium is not used to amplify DAB electron density. Moreover, immunolabeling also diminished with depth within the section and for that reason most of the sampled synapses show relatively weak DAB labeling, with DAB precipitate deposits mainly localized to membranes. Cryosubstituted materials were used for aspartate and glutamate colloidal gold immunolabeling because preliminary experiments demonstrated a higher sensitivity using this method compared to osmicated non-cryosubstituted sections embedded in hydrophobic resins like Epon-Araldite. These sections were processed in parallel and displayed more electron-dense calbindin-immunoreactivity (not shown). Thus, we used methods that traded strong calbindin-immunoreactivity for optimal detection of aspartate and glutamate immunoreactivities.

### Aspartate immunoreactivity

VAChT-IR terminals making synapses on calbindin-IR Renshaw cells consistently displayed colloidal gold aspartate-immunolabeling above neuropil staining. Aspartate-IR was observed in the axoplasm, over synaptic vesicle pools (Figs. 3A,C,D), and over mitochondria (see Fig. 3A inset). Sometimes we were able to localize immunogold particles inside synaptic vesicles docked in the presynaptic active zone (see Fig. 3D, inset), however more commonly colloidal gold was distributed to the general area with high numbres of synaptic vesicles being more difficult to resolve specific relations with individual synaptic vesicles. Serial sections through individual VAChT-IR synaptic boutons contained similar amounts of aspartate-IR (Figs. 3C,D). Calbindin-IR Renshaw cell dendrites and synaptic boutons (inhibitory) did not contain aspartate-IR (Fig. 3A,B).

To quantify aspartate-IR in VAChT-IR motor axon terminals, the area occupied by mitochondria was subtracted and colloidal gold particles were counted only outside mitochondria. Mitochondrial subtraction was intended to focus the analysis to synaptic vesicle pools and axoplasm regions from where synaptic vesicles could directly accumulate aspartate. Synaptic vesicle immunolabeling content could not be directly estimated because, with the exception of some occurrences as illustrated in figure 3D inset, indirect colloidal gold immunostaining using 10 nm particles absorbed to IgGs lacks enough resolution to determine whether immunoreactivities are located inside or outside synaptic vesicles of 30 to 50 nm in diameter (immunoreactive sites are estimated to localize within a 30 nm radius of the edge of the gold particle; [44]).

**Figure 5:**
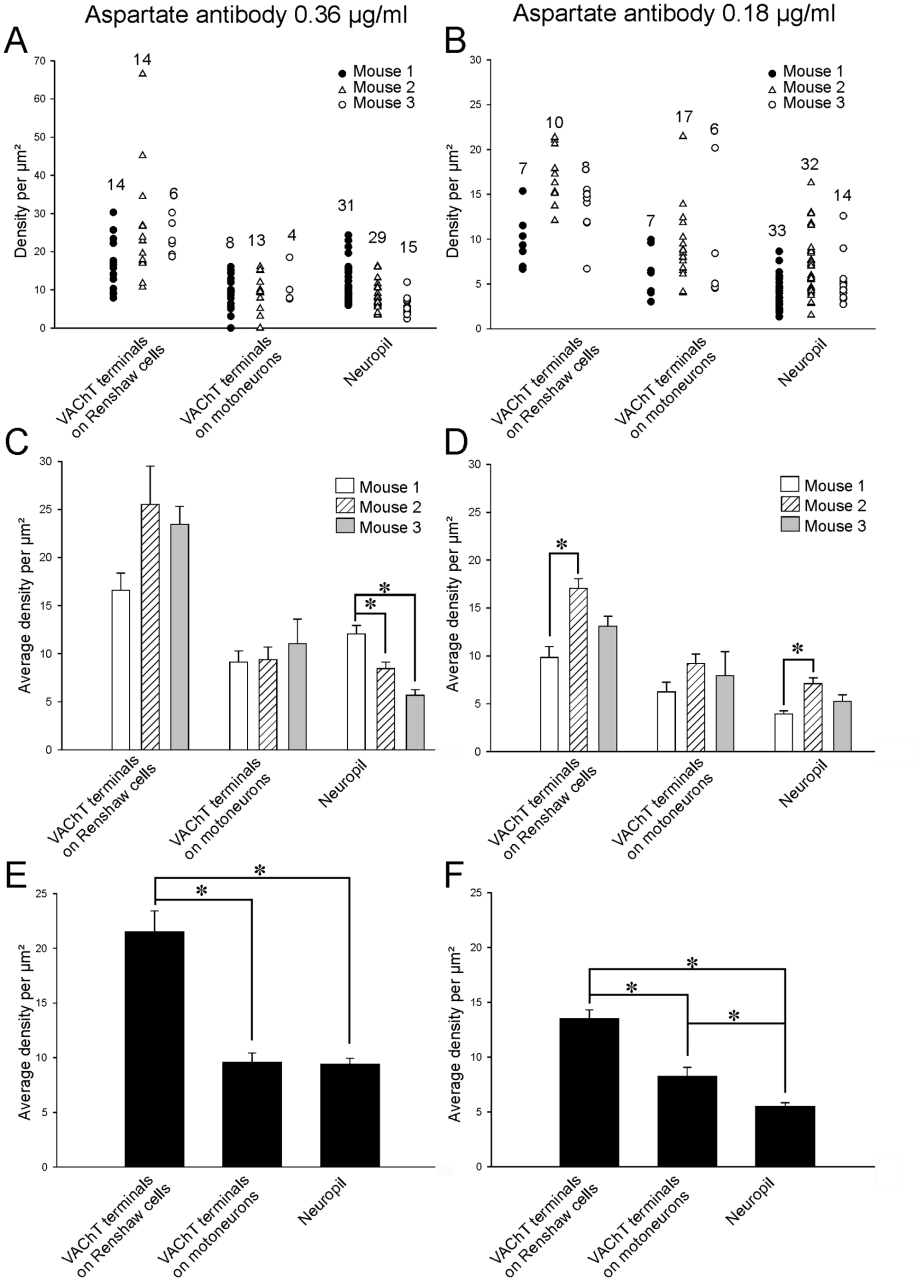
Quantification of aspartate-IR revealed enrichment over VAChT-IR motoneuron terminals contacting calbindin-IR Renshaw cells. **A, B**, Aspartate-IR gold densities in VAChT-IR boutons (on Renshaw cells or on motoneurons) and neuropil at antibody concentrations of 0.36 µg/ml (A) or 0.18 µg/ml (B). Estimates are separated according to animal sampled in A to D and pooled together for all animals in E and F. Each neuropil estimate is from an individual micrograph (2 to 4 areas analyzed per negative). The numbers of boutons or neuropil estimates are indicated above the respective plots. VAChT-IR boutons on Renshaw cells display aspartate-immunolabeling densities frequently higher than neuropil. VAChT-IR terminals contacting motoneurons contain immunolabeling densities usually similar to neuropil. C, D, Histograms showing average densities at high (C) or low (D) antibody concentrations for each type of terminal and neuropil in each animal. Error bars indicate ± SEM. At both antibody dilutions average aspartate-IR density in VAChT-IR boutons on Renshaw cells was consistently higher than neuropil averages or over VAChT-IR terminals contacting motoneurons. Variability among animals reached significance in two occasions using the low antibody concentration (asterisks in C and D; ANOVA, p<0.001 and post-hoc Tukey Test). E, F, Histograms representing pooled averages from all boutons sampled in the 3 animals at high (E) or low (F) antibody concentrations. Error bars also indicate ± SEM. At both dilutions the average density of aspartate-IR was significantly higher in VAChT-IR terminals contacting calbindin-IR Renshaw cells than in the neuropil or over VAChT-IR terminals contacting motoneurons (asterisks, p<0.001 ANOVA and post-hoc Tukey Tests). At the low antibody dilution C-boutons showed aspartate immunolabeling slightly above neuropil.

Colloidal gold aspartate-immunolabeling was estimated using low (0.18 µg/ml) and high (0.36 µg/ml) antibody concentrations to better determine possible differences with neuropil labeling. Thirty-four and 25 VAChT-IR motor axon boutons presynaptic to calbindin-IR Renshaw cell dendrites were analyzed respectively at each antibody concentration (Figs. 5A,B). Boutons were sampled from tissue blocks obtained from three different animals. Averaging the data from all boutons sampled in the three animals resulted in a colloidal gold average density inside VAChT-IR motor axon terminals that was significantly higher at 0.36 µg/ml antibody concentration than at 0.18 µg/ml (21.5 particles/µm^2^ ±1.9 ±SEM, vs. 13.8±0.2, p = 0.005, t-test) (Fig. 5E,F). Similarly, average neuropil labeling was significantly higher at 0.36 µg/ml antibody concentration compared to 0.18 µg/ml (9.3±0.6 vs. 5.5±0.4, p<0.001, t-test). Average aspartate immunolabeling inside VAChT-IR motor boutons was significantly greater than on the surrounding neuropil at both antibody dilutions and in each of the three animals analyzed (Figs. 5C,D) (p<0.001, ANOVA, post-hoc Tukey test p<0.001) or after combining all boutons and background estimates from all three animals (p<0.001, t-tests at each dilution)(Figs. 5E,F) Aspartate enrichment in VAChT-IR motor terminals relative to neuropil (labeling in VAChT-IR motor terminals/labeling in neuropil) was similar at both antibody concentrations (2.8× neuropil density ±0.8 SEM at 0.36 µg/ml and 2.5±0.03 SEM at 0.18 µg/ml; p = 0.31, t-test).

Although enrichment in aspartate immunolabeling inside VAChT-IR terminals compared to neuropil was detected in all three animals, absolute values of labeling intensity varied from animal to animal and from bouton to bouton. Inter-animal differences sometimes reached significance at the more diluted antibody concentration (p<0.05, ANOVA, Fig. 5D). We also noted parallel variability among animals in basal neuropil immunoreactivity (ANOVA, p<0.001) suggesting that the disparities are likely due to differential preservation of overall aspartate content during fixation and processing. Despite this variability, a similar proportion of VAChT-IR axon terminals were enriched with aspartate immunoreactivity. Pooling together all boutons, 80% and 71% of motor axon synaptic boutons, in respectively low and high antibody concentrations, displayed aspartate immunolabeling two standard deviations above the average neuropil labeling.

The density of aspartate-IR colloidal gold particles in VAChT-IR terminals contacting motoneurons (C-boutons) was consistently lower than in VAChT-IR motor axon terminals contacting Renshaw cells in all three animals and at both antibody concentrations (average densities in C-boutons were 9.6 particles/µm^2^ ±0.8 at high antibody concentration vs. 8.2±0.8 at low concentration; differences with motor axon terminals were always significant, p<0.001 ANOVAs, post-hoc Tukey test p<0.001) (Fig. 5E,F). Differences between C-boutons and neuropil immunolabeling were generally low but reached significance at the lower antibody concentration (8.2±0.8 vs. 5.5±0.4; p<0.001, t-test) (Fig. 5F). This difference was however inconsistent among samples obtained from different animals (Fig. 5D, p = 0.008 for animal 1; p = 0.06 for animal 2 and p = 0.178 for animal 3). Only a minority of VAChT-IR C-boutons, 17% for the low antibody concentration and 11% for the high, contained aspartate-immunogold density two standard deviations above their respective animal average neuropil labeling. C-boutons with aspartate labeling at this level were frequently outliers (Fig. 5A, B). Because of the unavoidable ultrastructural deterioration that occurs in these non-osmicated and Lowicryl-embedded triple immunolabeled sections, subsynaptic cisternae were not always unambiguously identified. Thus, it is possible that a few VAChT-IR contacts on motoneurons could originate from aspartate-enriched motor axons (see above). Nevertheless, the data do allow us to conclude that C-boutons, in general, do not contain aspartate immunolabeling at densities significantly above the neuropil. In summary, aspartate immunolabeling seems to be specifically enriched in cholinergic synapses originating from motor axons.

### Glutamate-immunoreactivity

Glutamate-IR densities were analyzed in the same manner, also using two antibody dilutions (0.17 µg/ml or 0.034 µg/ml) and in tissue sections from the same three animals. Immunolabeling above neuropil levels was noted in a portion of VAChT-IR synapses on calbindin-IR Renshaw cell dendrites and was more evident at the concentrated antibody dilution (Fig. 4A). Glutamate-IR enrichment was also frequently observed on vesicle pools of neighboring unlabeled Type I excitatory synapses. Inside these synapses, glutamate-IR usually appeared more intense than in VAChT-IR synapses (Fig. 4B). Renshaw cell inhibitory synapses labeled with DAB did not contain glutamate-IR colloidal gold above neuropil levels (Fig. 4C). In contrast to aspartate-IR, C-boutons frequently contained glutamate-IR at densities above neuropil (Fig. 4D).

**Figure 6:**
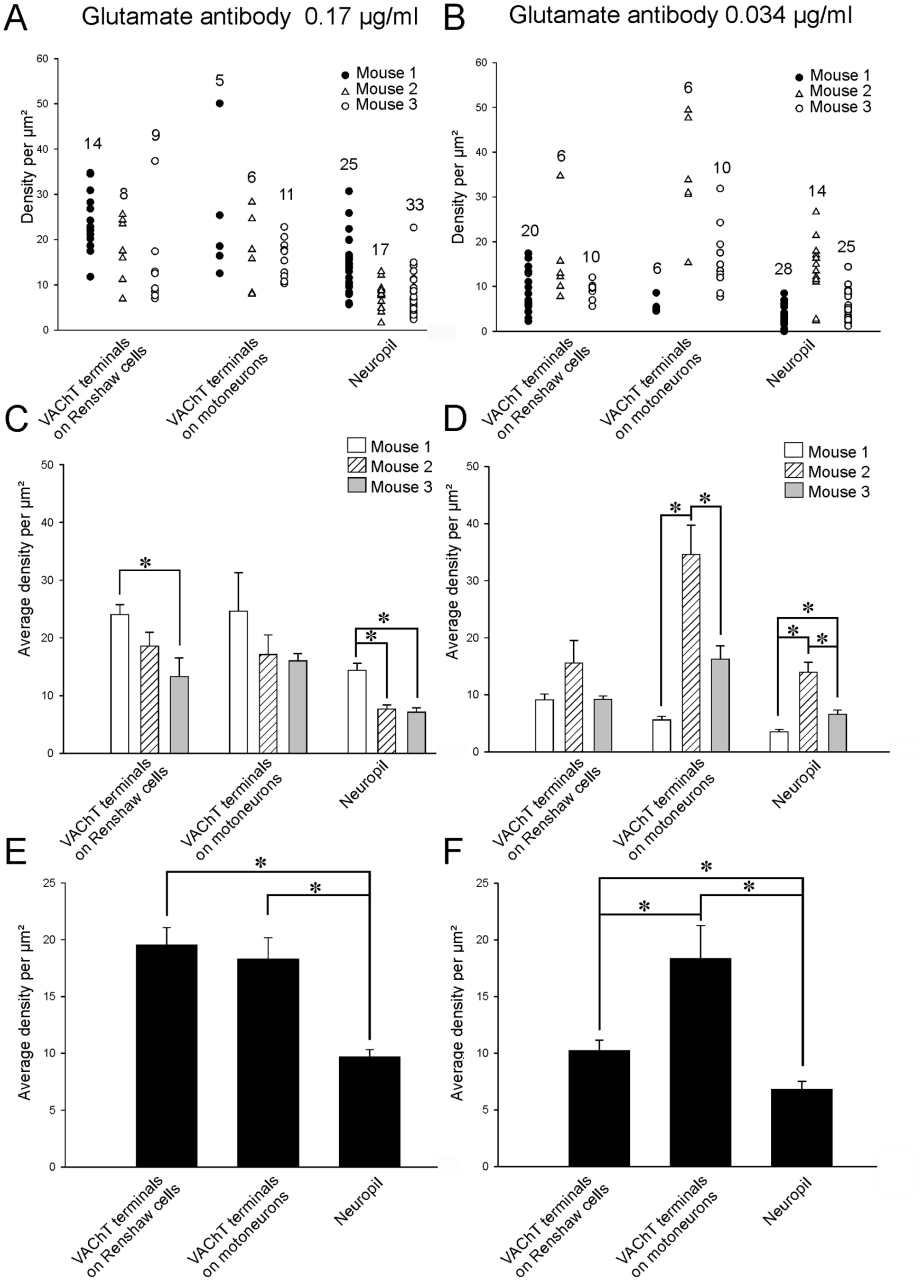
Quantification of glutamate-IR inside VAChT-IR motoneuron terminals contacting calbindin-IR Renshaw cells and C-boutons. **A, B**, Dot blots showing colloidal gold immunolabeling densities at high (A) or low (B) glutamate antibody concentrations in individual VAChT-IR motoneuron terminals contacting calbindin-IR Renshaw cells, in C-boutons (VAChT-IR terminals on motoneurons) and in the neuropil of the three experimental animals. C, D, Average density of glutamate-IR in each type of synaptic bouton and in the neuropil for each antibody concentration and animal analyzed. Inter-animal variability in glutamate immunolabeling of VAChT-IR motor axon or C-boutons and in neuropil labeling estimates was relatively low using the high antibody concentration (C) but increased when using the low antibody concentration (D). Asterisks indicate significant differences. E, F, Pooled data average histograms from the 3 mice using glutamate antibodies at 0.17 µg/ml (E) and 0.034 µg/ml (F). Glutamate-IR in VAChT-IR terminals contacting calbindin-IR Renshaw cell dendrites was significantly higher compared to neuropil at both antibody dilutions (asterisk indicate p<0.001, ANOVA and p<0.05 post-hoc pair-wise comparisons using a Tukey test). C-boutons showed immunolabeling density similar to VAChT-IR motor axon terminals at high antibody dilutions but significantly higher glutamate immunolabeling density compared to neuropil with low antibody concentrations. In both cases they contained significantly higher colloidal gold densities than the neuropil.

Glutamate-IR colloidal gold labeling density was estimated in 31 and 36 VAChT-IR motor axon boutons at respectively high and low antibody concentrations in tissues sampled from the same three animals (Fig. 6). Average glutamate-IR colloidal gold density was significantly higher with the more concentrated antibody (19.5±1.6 SEM compared to 10.2±0.9; p<0.01, Mann-Whitney Rank Sum test) (Fig. 6E,F). Average neuropil labeling varied in parallel with antibody concentration (9.7±0.7 at high antibody concentration and 6.8±0.7 at low antibody concentration). At high concentration there was some inter-animal variability in glutamate immunolabeling of VAChT-IR motor axon terminals or neuropil (Fig. 6C,D). Average glutamate-IR was, however, always significantly higher in VAChT-IR motor axon terminals compared to neuropil labeling in each animal (p<0.01, Mann-Whitney Rank Sum tests, Fig. 6C) or when pooling data from the three animals together (p<0.001, Mann-Whitney Rank Sum tests, Fig. 6E). At the lower antibody concentration, inter-animal differences in VAChT-IR motor axon terminals were not significant (Fig. 6D, p = 0.147, Kruskal-Wallis One Way Analysis of Variance on Ranks) but basal neuropil labeling was significantly different between animals (p<0.001 Kruskal-Wallis One Way Analysis of Variance on Ranks; p<0.05, post-hoc Dunn test for pair-wise comparisons). Pooled data from all three animals indicated that glutamate-IR inside VAChT-IR terminals was significantly higher than neuropil labeling even in low labeling conditions (p<0,001, Mann-Whitney Rank Sum test). At high and low antibody concentrations, average bouton labeling was respectively 2.0±0.2 and 1.7±0.5 times the neuropil staining. The enrichment values calculated at different antibody concentrations were not statistically significant (t-test, p = 0.214). Labeling was therefore consistently higher than neuropil, but average glutamate-IR enrichment from neuropil basal level was not as high as with aspartate immunolabeling. Analysis of scatter plots (Fig. 6A,B) suggests this was due to the presence of a large proportion of synapses with no labeling above neuropil in both high and low antibody dilutions. Consequently, only 44% and 21% of VAChT-IR motor axon terminals showed glutamate-IR two standard deviations above average neuropil labeling at high and low antibody concentrations respectively.

Compared to aspartate labeling, C-boutons were more consistently immunolabeled with glutamate antibodies. C-boutons displayed an average immunolabeling density significantly higher than neuropil at both glutamate antibody concentrations. Differences with neuropil labeling were always significant when making comparisons within animals (p<0.001 ANOVA, post-hoc Tukey tests p<0.05) or when pooling data from the three animals together (p<0.001, t-test). C-bouton glutamate labeling was similar to motor axon terminals at high antibody concentrations and even significantly higher at low antibody concentrations. Average enrichment levels from neuropil were similar at both dilutions (2.1±0.2 at the highest concentration of antibody and 2.2±0.3 at the lowest). About half of the C-boutons contained an immunolabeling density higher than two standard deviations from average neuropil labeling (54.5% at the highest antibody dilution, 45% at the lowest; n = 22 C-boutons analyzed at each dilution). In conclusion, glutamate is enriched with respect to neuropil in some VAChT-IR motor axons and C-boutons.

## Discussion

The major finding in this study is that motor axon synapses on Renshaw cells are enriched in both aspartate and glutamate, but at different levels. Aspartate was detected at 2.5 to 2.8 times background level and was consistently 2 standard deviations higher than the neuropil average in the majority of the terminals. Enrichment in glutamate immunoreactivity above 2 standard deviations of neuropil labeling was found in less than half of motor axon synapses on Renshaw cells and on average they contained 1.7 to 2 times the background levels. The average level of glutamate enrichment was similar to previous estimates using confocal microscopy [17]. From these data we conclude that aspartate is more highly enriched than glutamate in motor axon synapses on Renshaw cells.

### Mechanisms for the accumulation of glutamate and aspartate inside synaptic vesicles

The fact that aspartate and glutamate immunoreactivities are enriched in areas within synaptic boutons containing high density of synaptic vesicles, including presynaptic active zones, suggests that these amino acids could be accumulated in a releasable pool. In all of our analyses we substracted mitochondrial labeling to focus the study on potentially releasable pools of EAAs. A high immunoreactivity content in mitochondria of glutamate and aspartate, even also GABA, is well-known at many central synapses [37], [45], [46], [47]. They are best explained by their function as intermediate metabolites and their synthesis and storage during mitochondrial function, oxidative metabolism and the glutamine-glutamate-GABA cycle [48]. Since is unlikely that EAA mitochondrial content directly contributes to the releasable pools of these aminoacids, mitochondrial immunolabeling was not included in the estimates.

The exact mechanisms by which glutamate and aspartate are introduced inside motor axon synaptic vesicles remains unclear. The relatively lower levels of glutamate might agree with low expression of VGLUT2, but as reviewed in the introduction, the presence of VGLUT2 in motor axon terminals is at present not clear, with contradictory conclusions in current literature [17], [18], [20], [21]. All studies agree, however, that VGLUT2 and VAChT do not co-localize within intraspinal motor axon synaptic terminals, raising the possibility that cholinergic and “glutamatergic” motor axon synapses originate from either different motoneurons or different motor axon collaterals, as originally proposed by Herzog et al. [20]. The first possibility is highly unlikely because to our knowledge there are no non-cholinergic motoneuron phenotypes in the mammalian spinal cord. The second possibility will be an interesting exception to Dale's principle, but our data argue against it because we found significant enrichment of glutamate and aspartate inside VAChT-IR terminals. It is possible that by using VAChT immunoreactivity as the identification criterion, we might have missed a population of VAChT negative motor axon synaptic boutons more highly enriched in glutamate. However, we find this possibility also unlikely because bulk retrograde labeling of motor axons from ventral roots consistently failed to reveal motor axon synaptic varicosities lacking VAChT-immunoreactivity [17]. Present conclusions for motor axon synapses that are VGLUT2 positive *and* VAChT negative is based on two intracellular fills of motoneurons and their putative motor axons [18], [20]. A third study was not able to confirm this conclusion [21]. Differences in species and age (neonatal rodents vs. adult cats) might explain the discrepancy; but unfortunately the axonal nature of VGLUT2-IR structures in the two earlier studies was not fully established. Better and more complete labeling of motor axons and their intraspinal synapses would be necessary to unequivocally support the conclusion that different collaterals from single motor axons traffic distinct synaptic release machinery to different synaptic boutons.

An alternative possibility is that glutamate transport inside synaptic vesicles is mediated by a different protein, perhaps less efficient than VGLUTs. In addition, none of the known VGLUTs transport aspartate [26], [31], [32], [34], and therefore a transporter mechanism capable of carrying aspartate inside synaptic vesicles needs to be established for motoneurons. VGLUTs belong to the SLC17 family, members of this family include several Na^+^/phosphate co-transporters (SLC17A1-4), a lysosomal H^+^/sialic acid co-transporter (SLC17A5), the three VGLUTs (SLC17A6-8) and a vesicular nucleotide transporter (VNUTs) (SLC17A9) [49]. SLC17A5 was initially related to the transport of sialic acid in lysosomal membranes and associated with sialic acid storage hereditary diseases like Salla disease [50]. Its high amino acid sequence homology with VGLUTs (SLC17A6-8) led to SLC17A5 being tested as a possible aspartate transporter. The study of Miyaji et al., [51] concluded that SLC17A5 moves both aspartate and glutamate into synaptic vesicles of hippocampal synapses and pinealocytes, both known to release aspartate. However, a more recent study using SLC17A5 knockout animals demonstrated SLC17A5-independent uptake and release of aspartate in synaptosomal fractions and hippocampal synapses [33]. The levels of uptake and release of aspartate were unaltered in animals lacking SLC17A5, suggesting that loading of aspartate inside synaptic vesicles occurs using transporter mechanisms different from SLC17A5.

In agreement with these later results we failed to detect SLC17A5 immunoreactivity inside VAChT-IR synapses on Renshaw cells using three different commercial antibodies. These data were not presented in results because of doubts about the specificity of all the immunoreactivites detected by these antibodies in spinal cord sections. Each antibody was raised against different epitopes of the protein. One antibody was obtained from Abcam (cat#ab69819; lots# 598964, 827317; Cambrige, MA, USA) and was raised against amino acids 151 to 200 expanding regions in cytosolic and non-cytosolic regions of the molecule flanking the putative 4^th^ transmembrane domain. This region has 90% homology with amino acids 125 to 174 in the mouse sequence. The antibody obtained from Alpha Diagnostic International (cat.#SIAL11-A; lot# 4831P2; San Antonio, TX, USA) was raised against amino acids 84 to 99 in the first putative non-cytosolic domain between transmembrane regions 1 and 2 (i.e., extracellular if inserted in the plasma membrane or facing the lumen if inserted in the membrane of lysosomes or other cytoplasmic organelles). This region has a 93% identity with amino acids 54 to 68 in mouse SLC17A5. Finally, we also used an antibody obtained from Santa Cruz (V-18, SC50977; lot J2209) raised against a peptide (exact sequence proprietary Santa Cruz information) that is contained within amino acids 70 to 190 in the N-terminal. This antibody also resulted in the weakest immunolabeling of spinal cord sections. All three antibodies recognized SLC17A5 in heterologous expression systems, as indicated by manufacturer information. But in Western blots from spinal cord, in our hands, all three antibodies detected a band around the 51 to 60 kDa expected molecular weight of SLC17A5 and also several additional immunoreactive bands of higher and lower molecular weights (not shown). These were consistently present in many different conditions (denaturing, gel gradients, deglycosylation, protease inhibitor cocktails). Moreover, the immunoreactivities in spinal cord sections were different between all three antibodies. It is possible that all three antibodies are capable of immunodetecting SLC17A5, but each displays either different cross-reactivities with other proteins in tissue, or might detect SLC17A5 in different compartments depending on differential exposure or masking of their different epitopes. Similar specificity concerns with these antibodies were recently raised in another study [33]. Therefore, these antibodies rendered “dirty” immunoreactions in the spinal cord and require further investigation before the exact nature of SLC17A5 immunoreactivity can be reported with confidence. Nevertheless, we quantified the possible presence of SLC17A5 immunofluorescence in 131, 101 and 93 VAChT-IR synapses on calbindin-IR Renshaw cell dendrites with respectively the AbCam, Alpha Diagnostic and Santa Cruz antibodies. In only two cases we detected small immunofluorescence peaks inside VAChT-IR synapses that were above neuropil labeling. These results suggest that it is unlikely that SLC17A5 is present in motor axon synapses, but this conclusion needs to be confirmed with further antibodies that show better specificity and sensitivity of immunodetection in tissue. The transporter mechanism that accumulates aspartate, and maybe glutamate, inside synaptic vesicles in motor axons is therefore not yet settled and requires further investigation.

### Functional significance

Independent of the exact transport mechanism introducing aspartate and/or glutamate inside vesicles, motor axon evoked EPSPs and EPSCs in Renshaw cells display simultaneous postsynaptic activation of nicotinic, AMPA and NMDA receptors [15]–[18]. While NMDA receptors can be equally activated by released aspartate or glutamate, AMPA receptors can only be activated by glutamate and perhaps the low levels of glutamate inside motor axon boutons explains the smaller and more variable characteristics of AMPAergic currents at this synapse [15]. Thus, motor axon synapses on Renshaw cells can be considered to operate principally as nicotinic/NMDA synapses lacking strong AMPA currents, which implies that the nicotinic current is the principal mechanism removing the voltage-dependent Mg^2+^ block of the NMDA receptor [15], [16]. The necessity of close spatial and temporal activation of nicotinic and NMDA receptors therefore also argues against an organization in which different motor axon collaterals independently release acetylcholine and EAAs. Indeed, to increase the efficiency of NMDA activation it would be optimal if all neurotransmitters were co-released from the same vesicles; unfortunately, synaptic vesicle content could not be directly estimated because indirect colloidal gold immunostaining using 10 nm particles absorbed to IgGs lacks enough resolution to determine whether immunoreactivities are located inside or outside synaptic vesicles of 30 to 50 nm in diameter (immunoreactive sites are estimated to localize within a 30 nm radius of the edge of the gold particle [44]). Single vesicle co-release of glutamate and acetylcholine has been demonstrated from interneuron synapses in the tadpole spinal cord [52]. However, a recent preliminary report suggests that this is not the situation at motor axon synapses on Renshaw cells and concludes that acetylcholine and EAAs are released by different pools of synaptic vesicles [53]. If confirmed, this organization implies the need for synchronous “multiquantal” release to obtain a close spatial and temporal postsynaptic activation of nicotinic and NMDA receptors. Multiquantal release could occur from a single bouton or from adjacent boutons. The structure of the motor axon input on Renshaw cells could facilitate the latter interaction because single motor axon collaterals form several adjacent “en passant” synapses on small dendrite segments [54] and ultrastruturally is common to find many closely spaced synapses (see Figures 3A and 5A). This synaptic architecture, coupled with the fact that neurotransmitter spill-over at these synapses is common [16], could facilitate effective co-activation of nicotinic and NMDA receptors, even if the cognate neurotransmitters are released at different but closely-spaced synapses along single axon collateral branches.

The possibility that aspartate actions on NMDA receptors are responsible for a significant share of non-cholinergic effects of motoneurons inside the spinal cord implies that aspartate responsive interneurons expressing NMDA receptors will be preferentially targeted. One example is the elongation of Renshaw cell firing in response to motor axon inputs through NMDA-dependent mechanisms blocked by APV [15]. Another is the ability of ventral roots to activate the spinal cord Central Pattern Generator (CPG) and elicit rhythmic activity also through an APV sensitive mechanism [17]. This does not exclude additional actions of motor axon released glutamate since AMPA activation also occurs on Renshaw cells and more recent data suggest that entrainment of the spinal CPG by ventral root stimulation is sensitive to antagonists specific of AMPA activation of mGluR1 metabotropic glutamate receptors [55].

In conclusion, the immunocytochemical data presented here is consistent with the notion that motoneuron intraspinal synapses release acetylcholine, aspartate and to lesser extent glutamate and this fits with the pharmacological profiles of known synaptic actions of motor axons on Renshaw cells and the spinal cord network in general.
